# Population pharmacokinetic modeling of paired plasma–breast milk lamivudine data for estimation of infant exposure in breastfeeding mother–infant pairs

**DOI:** 10.1002/psp4.13274

**Published:** 2024-11-07

**Authors:** Francis Williams Ojara, Aida N. Kawuma, Shadia Nakalema, Isabella Kyohairwe, Ritah Nakijoba, Mohammed Lamorde, Henry Pertinez, Saye Khoo, Catriona Waitt

**Affiliations:** ^1^ Infectious Diseases Institute Makerere University College of Health Sciences Kampala Uganda; ^2^ Department of Pharmacology and Therapeutics Gulu University Gulu Uganda; ^3^ Department of Pharmacology and Therapeutics University of Liverpool Liverpool UK

## Abstract

Around 1.2 million women living with HIV give birth annually, majority of whom will breastfeed their infants while receiving antiretroviral therapy (ART). Lamivudine, a component of first‐line ART regimens crosses from maternal plasma to breast milk, with measurable concentrations in some breastfed infants. Wide variability in plasma‐to‐breast milk transfer has been reported within‐ or across studies, probably due to differences in sampling framework. This work sought to characterize the milk‐to‐plasma transfer of lamivudine, quantify inter‐patient variability and associated factors, and predict exposure of a breastfed infant. We explored data from an observational pharmacokinetic study that included 35 Ugandan mothers and their infants. Mothers received lamivudine doses of 150 mg twice daily or 300 mg once daily as part of their antiretroviral regimen. Pharmacokinetic sampling was undertaken across two visits approximately 8 weeks apart, providing 248 maternal plasma, 256 breast milk‐, and 151 infant blood concentrations, measured across a 24‐h sampling interval. A one‐compartmental model best described the plasma disposition of lamivudine, with first‐order absorption, interindividual variability on clearance and volume of distribution, and a proportional residual error model. A lag in time of plasma‐to‐breast milk drug accumulation was described using an effect compartment model with a milk‐to‐plasma ratio of 1.77. An estimated daily infant dose of 179.3 μg/kg (range: 125.8, 282.3) closely predicted the observed infant steady‐state concentrations and translated into 3.34% (2.13, 7.20) and 3.35% (1.10, 7.15) of the standard daily maternal dose in visits 1 and 2, respectively. The established modeling framework can be extended to other drugs.

**Study Highlights**

**WHAT IS THE CURRENT KNOWLEDGE ON THE TOPIC?**

A significant proportion of women breastfeed their infants while receiving lamivudine‐based antiretroviral therapy leading to infant exposure to the medication. Current data does not describe the plasma and breast milk profiles of lamivudine across the entire dosing interval limiting accurate characterization of breast milk transfer and estimation of infant exposure.

**WHAT QUESTION DID THIS STUDY ADDRESS?**

Can we characterize changes in plasma and breast milk lamivudine concentrations across time from paired maternal plasma–breast milk concentration data to enable quantification of plasma‐to‐breast milk transfer across the entire dosing interval and estimation of infant exposure?

**WHAT DOES THIS STUDY ADD TO OUR KNOWLEDGE?**

A one‐compartmental plasma disposition model coupled with an effect compartment model adequately characterized maternal plasma‐to‐breast milk accumulation of lamivudine. The model predicts consistently low resultant infant daily lamivudine exposure, within range with observed infant plasma concentrations.

**HOW MIGHT THIS CHANGE DRUG DISCOVERY, DEVELOPMENT AND/OR THERAPEUTICS?**

The developed framework can be used to characterize the plasma‐to‐breast milk transfer of other drugs across therapeutic areas.

## INTRODUCTION

Lamivudine, a nucleoside reverse transcriptase inhibitor, is a component of World Health Organization‐recommended first‐line antiretroviral regimens and for over two decades the drug has been used in the treatment of HIV and hepatitis B.^1^ Lamivudine is used across diverse populations including adults, breastfeeding women, and infants owing to its good efficacy and safety profile.^1^, ^2^ An estimated 1.2 million pregnant women were living with HIV globally in the year 2022,^3^ a significant proportion of whom received lamivudine‐based antiretroviral regimens. A combination of tenofovir, lamivudine, and dolutegravir constitutes the current first‐line option among adults and adolescents living with HIV, with dolutegravir replacing the former first‐line option of efavirenz.^4^ A dose of lamivudine of 2 mg/kg twice daily is recommended in infants <4 weeks of age increasing to 4 mg/kg twice daily in those ≥4 weeks.^5^

Lamivudine has been shown to cross from maternal plasma to breast milk with high concentrations measured in breast milk.^6^, ^7^, ^8^, ^9^, ^10^, ^11^ As such, breast milk is a notable route of lamivudine exposure for breastfed infants.^8^, ^9^, ^10^, ^11^ Infant drug exposure is affected by several factors, including the drug's physicochemical properties, maternal physiology, infant feeding patterns relative to the time of maternal dosing, and infant physiology,^12^ most of which are not explicitly quantified in lactation pharmacokinetic studies. Evidence from the pre‐universal ART era when short course options for prevention of mother to child transmission of HIV were used, indicates a high risk of the infant developing multiclass HIV drug resistance after acquiring HIV during breastfeeding when the mother was on ART due to the selective pressure of low concentrations of drug around IC90.^13^, ^14^, ^15^

Plasma‐to‐breast milk transfer of lamivudine has widely been described based on paired maternal plasma–breast milk data obtained at diverse sampling time‐points across the maternal dosing interval.^7^, ^8^, ^9^, ^10^, ^11^, ^16^ Studies report wide intra‐ and inter‐study variability in plasma‐to‐breast milk transfer of lamivudine.^17^ Studies that performed longitudinal intensive plasma and breast milk concentration measurements reported slower elimination of lamivudine from breast milk resulting into increased milk‐to‐plasma ratio across the dosing interval, which was not apparent in studies with sparsely sampled plasma and breast milk data.^17^ Plasma concentrations of lamivudine in breastfeeding infants ranged between 2 and 6% of the maternal plasma concentrations and declined across the post‐partum period.^17^ Population pharmacokinetic modeling characterizes population‐ and individual‐level changes in drug concentrations across time for effective longitudinal comparison of breast milk and plasma drug exposure.^18^

We aimed to (1) develop a mechanistic population pharmacokinetic model characterizing the longitudinal transfer of lamivudine from maternal plasma‐to‐breast milk at steady‐state, (2) evaluate the impact of different maternal demographic and clinical covariates on maternal plasma‐to‐breast milk transfer of lamivudine, (3) estimate infant exposure to lamivudine through breast milk, and (4) compare the estimated infant exposure with measured infant lamivudine concentrations.

## METHODS

### Study dataset

The dataset used in this analysis was obtained from an observational study whose main objective was characterizing the pharmacokinetic transfer of first‐line antiretroviral drugs, efavirenz, tenofovir, and lamivudine, from maternal plasma‐to‐breast milk and the subsequent pharmacokinetic exposure among breastfed infants. Briefly, a cohort of 35 mothers living with HIV and receiving (or who were scheduled to commence) first‐line antiretroviral treatment were recruited antenatally at the Infectious Diseases Institute (IDI) and affiliated clinics in Kampala (Uganda) between 2016 and 2017. Ten mothers received lamivudine 150 mg twice daily (12‐hourly), while 25 received 300 mg once daily (24‐hourly), in combination with nevirapine and zidovudine, or efavirenz and tenofovir disproxil fumarate, respectively, which were the recommended first‐line regimens for use in pregnancy and postpartum at the time of the study. Mothers were allocated in equal numbers to attend two out of three potential study visits at 1–2 weeks; 4–6 week; and 10–12 weeks post‐partum.

In 14 mothers who routinely took their medication in the morning, plasma and breast milk samples were collected at pre‐dose (0 h), 1, 2, 4, and 8 h post‐dose, and at pre‐dose (0 h), 2, 4, and 8 h post‐dose, respectively, relative to directly observed dosing. Infant blood was collected at maternal pre‐dose, 4 h, and 5–8 h post maternal dose, with the aim of capturing an infant maximum and “trough” concentrations. In 21 mothers who routinely took their medication in the evening, paired plasma and breast milk samples were collected at 12, 16, and 20 h post self‐reported‐dose, while infant blood was sampled at 12 h, and at a random time during the day. Mothers were advised to freely breast feed, and breast milk was sampled relative to the time of last maternal dose by manual expression of 1 mL. Maternal dried blood spots were prepared by accurately spotting 50 μL of blood from an EDTA tube onto the Whatman 903 Protein Saver card, and dried breast milk spots were prepared by spotting 30 μL aliquots of breast milk onto each circle on the Whatman 903 Protein Saver card. Infant dried blood spots were prepared by directly spotting from a heel prick sample onto the card. All samples were transported ambiently to the Department of Molecular and Clinical Pharmacology, University of Liverpool, UK, for analysis. Mothers freely breastfed their infants at all times during the study. Concentrations were quantified using a validated LC–MS/MS assay with an analytical lower limit of quantification of 5 ng/mL for plasma and 16.6 ng/mL for breast milk.^11^ A correction factor previously reported in Waitt et al.,^11^ was used to convert the infant DBS to plasma concentrations.

Additionally, different maternal and infant clinical and demographic characteristics were documented (Table 1). The study was approved by the University of Liverpool and the Joint Clinical Research Center (JCRC, Kampala‐Uganda) ethics committees, and registered with the Uganda National Council of Science and Technology.

**TABLE 1:** Maternal and infant characteristics from the study characterizing maternal plasma‐to‐breast milk transfer of lamivudine.

Patient characteristics	Median (range)
Maternal weight (kg)	64.0 (50.0, 89.0)
Maternal height (cm)	161 (148, 172)
Maternal age (years)	30 (19, 40)
BMI (kg/m^2^)	24.8 (20.0, 30.5)
CRCL (mL/min)	134.6 (89.2, 184.7)
Visit 1 (Days postpartum)	13.0 (7.00, 43.0)
Visit 2 (Days postpartum)	73.5 (36.0, 84.0)
Infant weight–Visit 1 (kg)	3.60 (2.40, 5.58)
Infant weight–Visit 2 (kg)	5.17 (3.90, 7.13)

Abbreviations: BMI, Body mass index; CRCL, Creatinine clearance; Infant weight–1, Infant weight corresponding to study visit 1; Infant weight–2, Infant weight corresponding to study visit 2 Visit 1, Study visit 1; Visit 2, Study visit 2.

### Population pharmacokinetic analysis of plasma and breast milk lamivudine concentrations

Maternal plasma‐to‐breast milk transfer of lamivudine was characterized in two steps. Firstly, the one‐ and two‐compartmental plasma disposition models were evaluated to characterize the plasma disposition of lamivudine. An absorption lag time and transit absorption compartments were evaluated to characterize the observed delay in oral absorption of lamivudine. Interindividual variability was evaluated on all model parameters using an exponential model. Residual unexplained variability on lamivudine concentrations was modeled using the additive, proportional, or combined additive and proportional models.

Maternal weight, age, creatinine clearance predicted from serum creatinine using the Cockcroft and Gault formular^19^ and body mass index (BMI) were evaluated as covariates on clearance, whereas BMI and weight were evaluated as covariates on the volume of distribution of lamivudine. A stepwise covariate modeling (SCM) approach^20^ was adopted to evaluate the impact of selected covariates on lamivudine disposition. A less stringent *p*‐value (<0.05) was used in the forward inclusion step of the SCM, followed by a more stringent *p*‐value (<0.01) in the backward elimination step. Additional criteria for covariate retention were based on comparison of the precision of parameter estimates. Linear, exponential, and power parameter–covariate functional relationships were evaluated, and covariate effects were measured with the covariates centered on the median covariate value.

**FIGURE 1:**
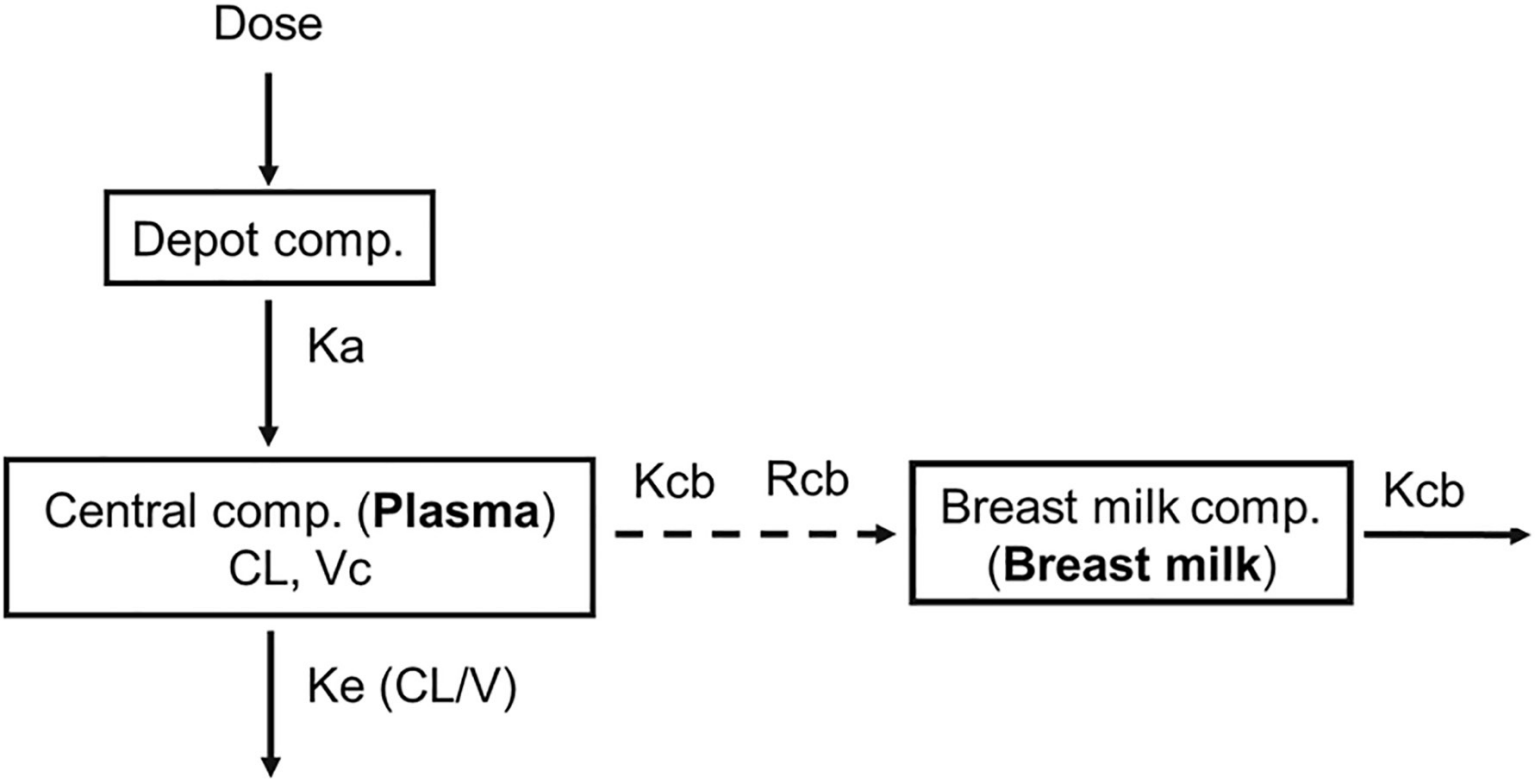
A one‐compartmental plasma pharmacokinetic model, linked to a breast milk “effect compartment,” to characterize the lactation pharmacokinetics of lamivudine. Ka, 1st–order absorption rate constant; CL, Plasma clearance; Vc, Volume of distribution of the central compartment; Ke, Elimination rate constant from the central compartment; Kcb, Plasma and breast milk equilibration rate constant characterizing the time delay in transfer of lamivudine from plasma to breast milk; Rcb, Lamivudine breast milk accumulation rate constant characterizing the milk‐to‐plasma ratio.

Second, the transfer of lamivudine from maternal plasma to breast milk in mothers with paired plasma and breast milk samples was characterized using an effect compartment model as previously described by Court et al.^18^ This model, Equations (1), (2), (3), described the breast milk‐to‐plasma (M:P) accumulation ratio and the time delay in transfer from maternal plasma to breast milk (Figure 1). The postpartum day of PK sampling (visit day) was evaluated as a covariate on the milk‐to‐plasma ratio.

(1) $$\frac{\mathrm{dA}1}{dt}=-\mathrm{Ka}*A1$$

(2) $$\frac{\mathrm{dA}2}{dt\;}=\mathrm{Ka}*A1-\frac{\mathrm{CL}}{\mathrm{VC}}*A2$$

(3) $$\frac{\mathrm{dA}3}{dt\;}=\mathrm{Kcb}*\left(\mathrm{Rcb}*\frac{A2}{\mathrm{VC}}-A3\right)$$

whereby A1 and A2: amounts of lamivudine in the depot and plasma compartments, respectively, A3: concentration of lamivudine in the breast milk compartment, respectively; Ka: 1st – order absorption rate constant; CL: Plasma clearance; VC: Volume of distribution of the central compartment; Kcb: plasma‐to‐breast milk equilibration rate constant characterizing the time delay in transfer of lamivudine from plasma to breast milk; Rcb: Lamivudine breast milk accumulation rate constant characterizing the milk‐to‐plasma ratio.

To visualize the impact of varying Rcb on plasma‐to‐breast milk transfer of lamivudine, concentrations–time profiles were deterministically simulated for the 150 mg 12‐hourly and 300 mg 24‐hourly dosing schedules at the model estimated typical (median), 5th and 95th percentiles of RCB (assuming a normal distribution of parameters), and typical parameter levels for the other model parameters (Ka, CL, VC, Kcb).

### Estimation of infant breast milk exposure to lamivudine

The daily infant dose was estimated considering model estimated average breast milk concentrations, milk‐to‐plasma ratio (RCB), and an estimated daily infant breast milk intake of 0.15 L/kg/day^18^, ^21^ (Equations 4 and 5). The average breast milk concentration over 24 h was calculated from the average plasma concentrations over 24 h (Equation 4). Individual plasma AUC from time 0 to 24 were derived by integrating lamivudine concentrations over time, from 0 to 24 h, and the average concentration over 24 h calculated by dividing the AUC 0–24 h by 24.

(4) $${\text{Conc}}_{\text{Milk}}=\mathrm{Rcb}•{\text{Conc}}_{\mathrm{Ave}}$$

(5) $${\text{Dose}}_{\text{infant}}={\text{Conc}}_{\text{Milk}}•{\mathrm{Vol}}_{\text{Milk}}$$

whereby Rcb: Milk‐to‐plasma ratio of lamivudine; ${\text{Conc}}_{\mathrm{Ave}}$: Average maternal plasma concentration; ${\text{Conc}}_{\text{Milk}}$: lamivudine concentration in breast milk and ${\mathrm{Vol}}_{\text{Milk}}$: volume of breast milk ingested by a lactating infant.

The relative infant dose (RID) was calculated from the estimated daily infant dose and the maternal daily dose using Equation 6:

(6) $$\text{Relative Infant Dose}\;\left(\%\right)=\frac{\;{\text{Dose}}_{\text{infant}}\left(\mathrm{mg}/\mathrm{kg}/\mathrm{Day}\right)\;}{\text{Maternal Dose}\;\left(\mathrm{mg}/\mathrm{kg}/\mathrm{Day}\right)\;}•100$$

Furthermore, verification of whether the estimated daily infant lamivudine doses predicted the measured infant plasma concentrations was undertaken. Infant apparent plasma clearance was derived from a pediatric lamivudine plasma disposition model accounting for infant weight and age^22^ (Equation 7) and the infant steady‐state plasma concentration was calculated using Equation 8.

(7) $$\frac{\mathrm{CL}}{F}\left(Lh^{-1}\right)=12.7*{\left(\frac{\mathrm{WT}}{7}\right)}^{0.75}*\frac{{\mathrm{Age}}^{1.47}}{0.25^{1.47}+{\mathrm{Age}}^{1.47}}$$

whereby CL/F is the apparent clearance; WT (kg): total body weight and 7 kg is the population median weight, and Age (years): age since date of birth

(8) $$C_{\mathrm{ss}}=\frac{{\text{Dose}}_{\text{infant}}}{{\mathrm{CL}}_{\text{infant}}•\tau }$$

whereby $C_{\mathrm{ss}}$ is the estimated infant steady‐state concentration; ${\text{Dose}}_{\text{infant}}$ is the infant dose; ${\mathrm{CL}}_{\text{infant}}$: is the estimated infant clearance and τ: is the dosing interval of 24 h.

### Model evaluation

Comparison of nested models employed the likelihood ratio test: a decrease in the objective function value (ΔOFV) ≥3.84 indicating statistical significance of the larger to the smaller model, given *α* = 0.05 and one additional parameter.^23^ Additionally, precision of parameter estimates and simulation‐based visual predictive checks (VPCs) were compared for different models.^24^ To calculate parameter precision, multiple (*n* = 1000) bootstrap datasets were generated by randomly drawing, with replacement, from the original dataset followed by evaluation of the final model.^23^

### Software

Dataset preparation and statistical analysis were performed in R (4.2.3),^25^ whereas pharmacokinetic modeling was performed using the first‐order conditional estimation with interaction method in NONMEM 7.4.3 with assistance of PsN (4.2.0)^26^ and Pirana (21.11.1).^27^

## RESULTS

### Study dataset

**FIGURE 2:**
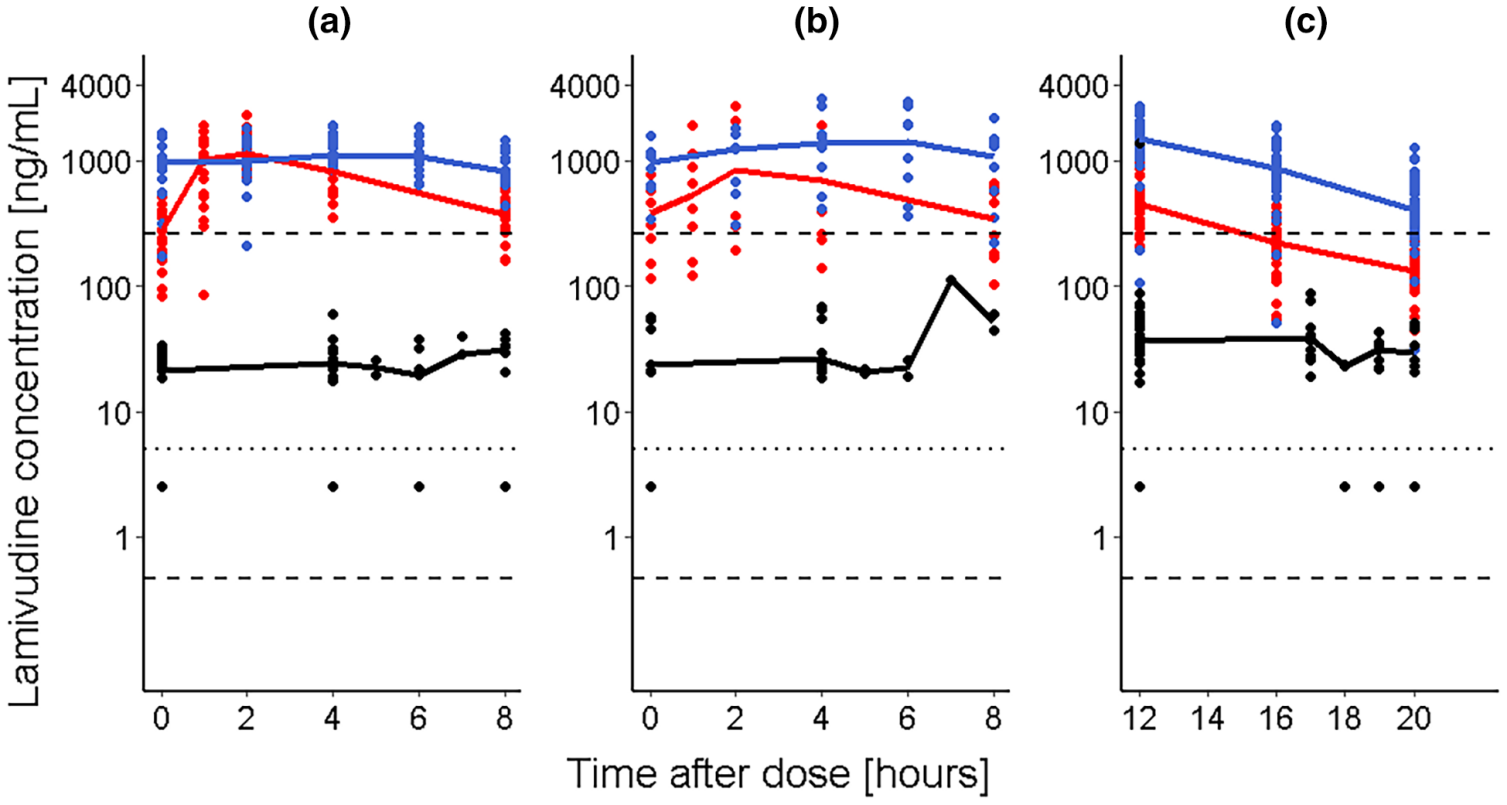
Pooled lamivudine concentration‐time profiles across different matrices. Solid circles represent concentrations, red: Maternal plasma; blue: Maternal breast milk; black: Infant blood, solid lines represent average of drug concentration across time, red: Maternal plasma; blue: Maternal breast milk; black: Infant blood; dashed lines: Lamivudine half maximum inhibitory concentration ranges for HIV‐1 virus (IC50, 0.458, 261.3 ng/mL)^1^; dotted line: Lower limit of quantification (LLOQ, 5 ng/mL). Panels (a) Patients treated with 150 mg 12‐hourly; (b) Patients treated with 300 mg 24‐hourly and sampled 0–8 h; (c) Patients treated with 300 mg 24‐hourly and sampled 12–20 h.

The mothers were generally young, with a median normal body weight and elevated creatinine clearance values (Table 1). The timepoints for study visits 1 and 2 were, median (range, in days), 13.0 (7.0, 43.0) and 74.0 (36.0, 84.0), respectively. Overall, 248 maternal plasma lamivudine concentrations, 256 breast milk concentrations, and 151 infant plasma concentrations were measured across all pharmacokinetic sampling timepoints. The longitudinal lamivudine plasma concentration‐time profiles from the 150 mg 12‐hourly dosing was in the same ranges as that for the 300 mg 24‐hourly dosing. The median infant concentrations (range) were 35.2 (2.84, 1563.9) ng/mL and 26.7 (2.84, 78.5) ng/mL in visits 1 and 2, respectively, with one outlier, extremely high value (1563.9 ng/mL) in visit 1. The infant concentration–time profiles were fairly constant across time and significantly lower than both the maternal plasma and breast milk concentrations (Figure 2). Twenty‐five (17.0%) infant blood concentrations were below the LLOQ, with 15 (60%) in the 150 mg 12‐hourly maternal dosing group, and with higher proportions (47.0%) at maternal pre‐dose times. No specific trends in distribution of below LLOQ samples were observed in infants with mothers in the 300 mg 24‐hourly dosing schedule. Infant below LLOQ samples were set to 2.5 ng/mL (50% of the LLOQ) to enable numeric summary. The plasma concentrations peaked earlier than the breast milk concentrations (2 h vs. 6 h) signifying differences in distribution and elimination processes in the two compartments.

### Population pharmacokinetic analysis of plasma and breast milk lamivudine concentrations

The plasma disposition of lamivudine was adequately characterized by a one‐compartmental plasma disposition model (Table 2). A simple first‐order process adequately described the systemic drug absorption compared to more complex absorption lag time model (ΔOFV < 3.84) and transit absorption models (estimations unsuccessful). Interindividual variability was estimated on plasma clearance, volume of distribution, and the milk‐to‐plasma ratio. A proportional error model best characterized the residual unexplained variability on plasma concentrations.

**TABLE 2:** Parameter estimates of the population pharmacokinetic model characterizing the maternal plasma to breast milk transfer of lamivudine.

Parameter (Units)	Estimates (%RSE)	Bootstrap median (95% CI)
CL (L•h^−1^)	19.4 (4.0)	19.5 (17.5–21.5)
VC (L)	184 (7.00)	180.1 (122.2–279.9)
Ka (h^−1^)	1.87 (19.0)	1.85 (1.25–2.53)
Kcb (h^−1^)	0.245 (12.0)	0.251 (0.185–0.311)
Rcb	1.77 (6.00)	1.76 (1.58–1.95)
IIV CL, % CV	20.9 (16.0)	20.2 (12.7–26.6)
IIV VC, % CV	76.4 (35.0)	71.5 (15.5–137)
IIV CL‐VC, % CV	42.0%	40.1
IIV Rcb, % CV	15.9 (19.0)	16.0 (9.50–21.5)
RUV, PROP, PLASMA, % CV	38.3 (7.7)	38.7 (33.2–43.5)
RUV, PROP, BM % CV	30.5 (8.6)	31.1 (25.3–36.7)

Abbreviations: CL, Plasma clearance; CV, coefficient of variation calculated as $100*\sqrt{\omega^{2}}$; IIV, Interindividual variability; Ka, 1st – order absorption rate constant; Kcb, plasma‐to‐breast milk equilibration rate constant characterizing the time delay in transfer of lamivudine from plasma to breast milk; Rcb, Lamivudine breast milk accumulation rate constant characterizing the milk‐to‐plasma ratio; RUV, Random unexplained variability; VC, Volume of distribution of the central compartment.

Model parameters were generally precisely estimated. The available data did not support estimation of interindividual variability on the absorption rate, resulting into a high parameter shrinkage (i.e., 75%), as such the value was fixed to zero. The covariate effects of creatinine clearance on clearance and weight on the volume of distribution were statistically significant after stepwise covariate analysis (ΔOFV = 35.6, *p*‐value <0.01). However, covariate effects were imprecisely estimated (%RSE > 30%), based on the standard errors from the variance–covariance matrix (Table S1). As such, the final plasma disposition model has no covariates included.

**FIGURE 3:**
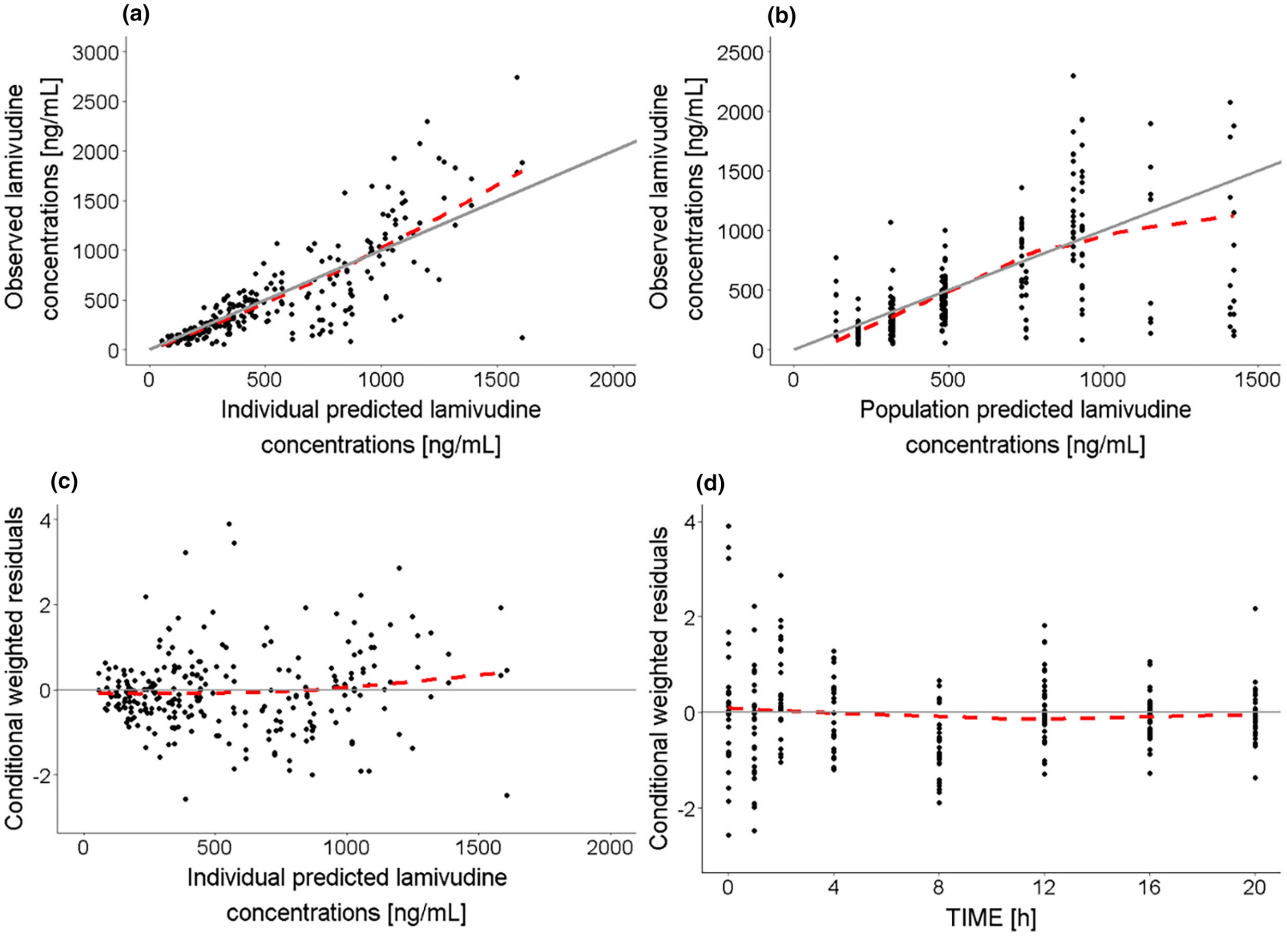
Goodness‐of‐fit for lamivudine plasma data from the population pharmacokinetic model characterizing the maternal plasma‐to‐breast milk transfer of lamivudine. Solid circles: Data points; solid gray line: Reference line; dashed red line: Trend line for the observed data; Plots (a) Observed versus individual predicted lamivudine concentrations; (b) Observed versus population predicted lamivudine concentrations; (c) Conditional weighted residuals versus individual predicted lamivudine concentrations; (d) Conditional weighted residuals versus time.

**FIGURE 4:**
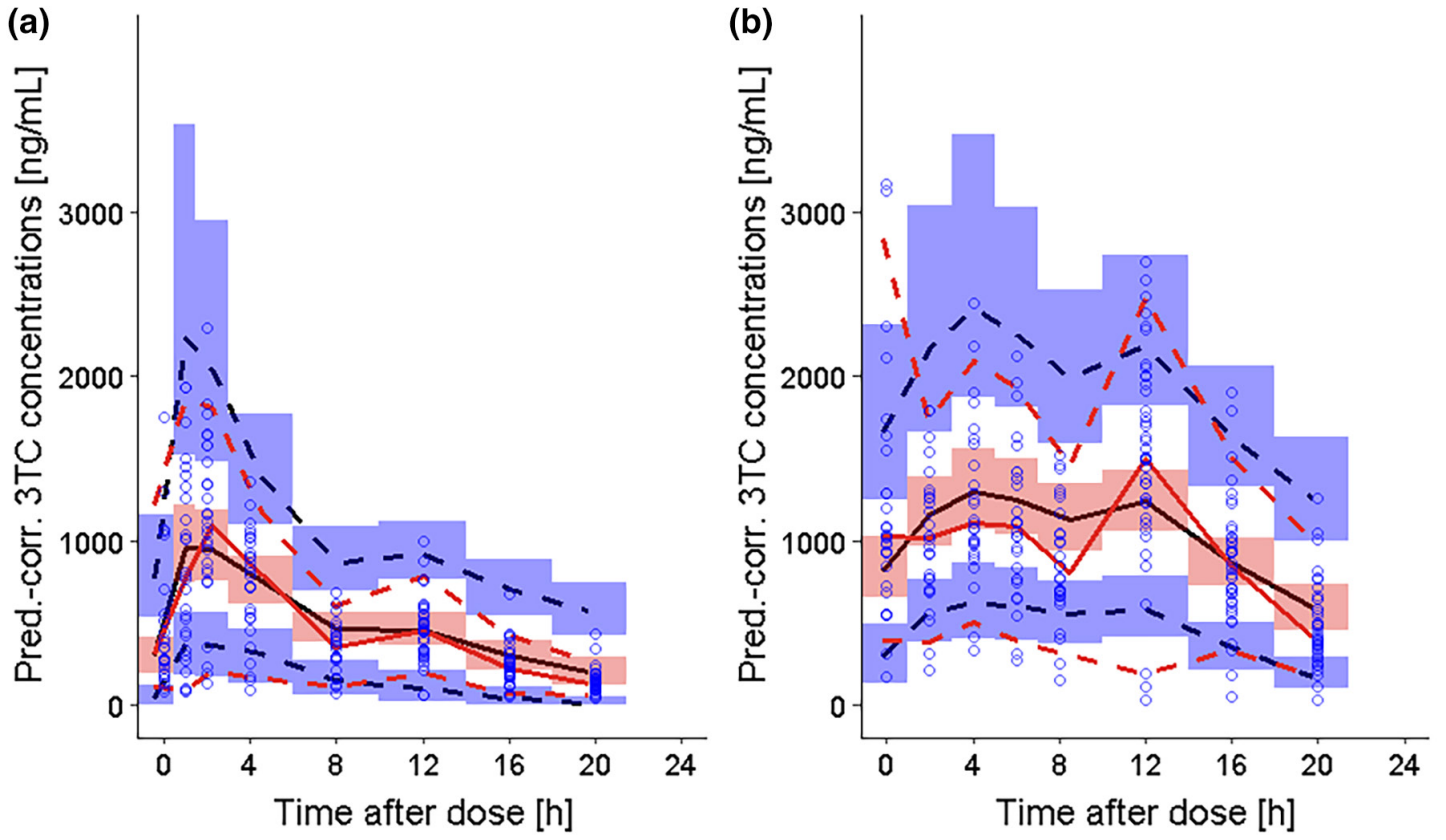
Prediction‐corrected visual predictive checks for plasma and breast milk lamivudine concentrations. Blue circles, observed lamivudine concentration data; black lines, median (solid), 5th, 95th percentiles (dashed) of lamivudine concentration data; red lines, median (solid), 5th, 95th percentiles (dashed) of model‐predicted concentrations; shaded areas, 90% confidence intervals of model‐predicted percentiles. Panels (a) lamivudine plasma concentration; (b) lamivudine breast milk concentration.

The plasma‐to‐breast milk transfer of lamivudine was adequately described with a milk‐to‐plasma ratio of 1.77 (Table 2). Inclusion of a correlation term between plasma clearance and the volume of distribution further improved model fit (ΔOFV > 3.84). A proportional error model best described the residual unexplained variability in breast milk concentrations. The postpartum day of PK sampling did not significantly affect the milk‐to‐plasma ratio. The final model demonstrated good fit for the observed plasma and breast milk lamivudine concentration data (Figures 3 and S1, respectively). The plots of individual‐ or population‐predicted plasma concentrations against observed concentrations (Figure 3a,b, respectively) symmetrically distributed around the reference line and the plots of conditional weighted residual against individual predictions (Figure 3c) and against time (Figure 3d) showed a symmetrical distribution around the reference line. The VPC for model evaluation showed that the model adequately predicted the median plasma lamivudine concentration–time profile but over‐predicted higher concentrations (Figure 4).

**FIGURE 5:**
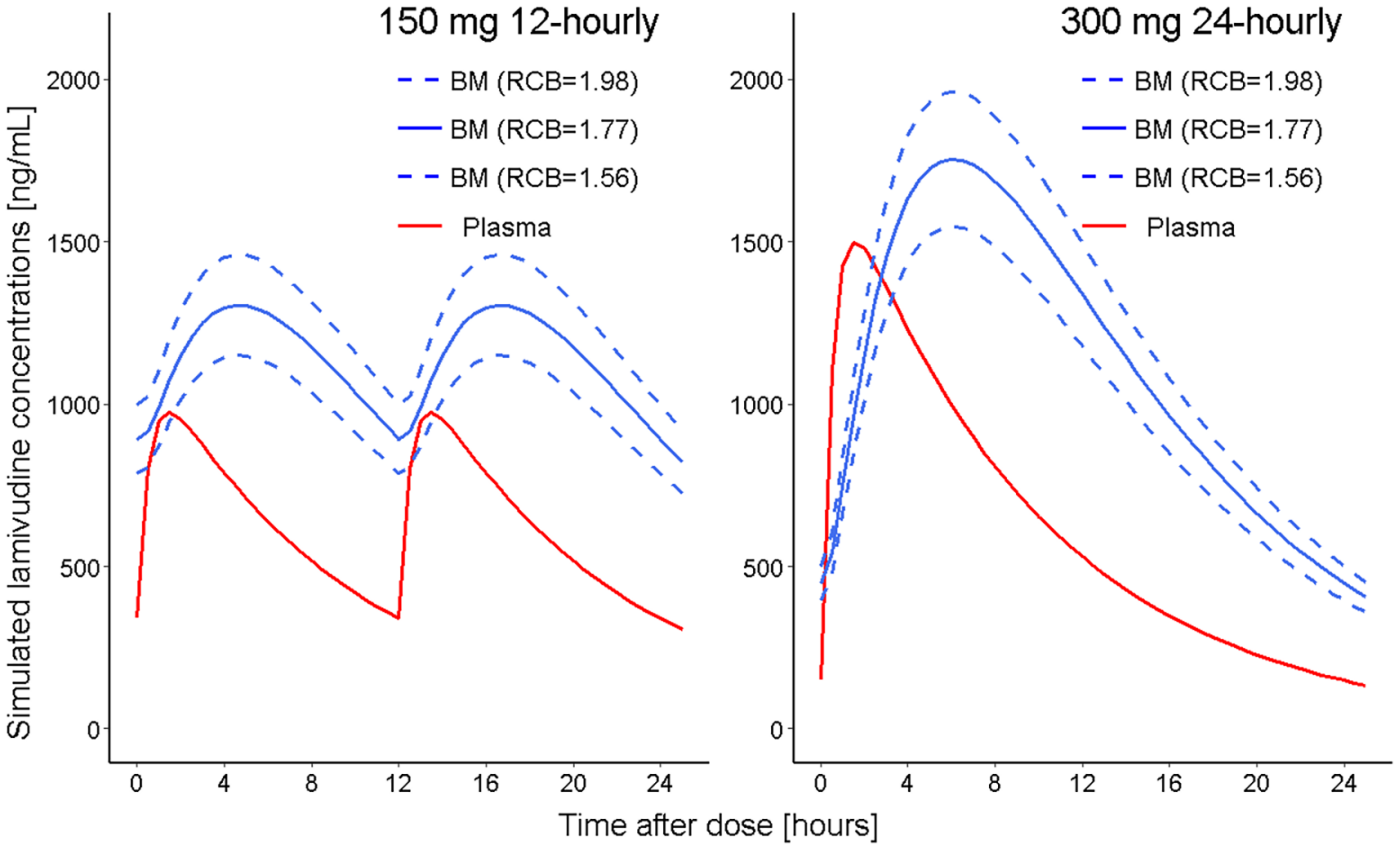
Plasma and breast milk lamivudine concentration‐time profiles from simulations using different milk‐to‐plasma ratio parameter (Rcb) levels. Lines, solid red: Typical plasma concentrations derived from the typical (median) model estimated parameters; solid blue: Typical breast milk concentrations derived from typical (median) model estimated parameters; dashed blue: Breast milk concentrations corresponding to the 5th percentile (lower) and 95th percentile (higher) of the milk‐to‐plasma ratio parameter i.e. Rcb = 1.56 and 1.98, respectively.

The observed delay in transfer from plasma to breast milk was well described by the model as demonstrated in the deterministic simulation based on the estimated model parameters (Figure 5). The deterministic simulation depicts maternal plasma and breast milk concentrations for a typical patient (median model parameters) receiving 150 mg of lamivudine 12‐hourly or 300 mg 24‐hourly, and explore profiles for the 5th and 95th percentiles of milk‐to‐plasma ratios.

### Estimation of infant breast milk exposure to lamivudine

The estimated daily lamivudine infant dose was median (range), 179.3 μg/kg (125.8, 282.3) in both visits 1 and 2. This translated into RID % of 3.34 (2.13, 7.20) in visit 1 and 3.35 (1.10, 7.15) in visit 2 and an estimated 2.26% (1.57, 3.53) of the recommended daily infant dose considering the 4 mg/kg twice daily recommended daily infant dose for HIV treatment in an infant ≥4 weeks but <3 months. The estimated average steady‐state infant plasma concentrations was 49.7 ng/mL (11.5, 178.1) in visit 1 and 10.5 (6.21, 23.0) in visit 2.

## DISCUSSION

We developed a lactation population pharmacokinetic model for lamivudine, using observational plasma and breast milk concentration data, to improve the characterization of infant lamivudine exposure through breast milk. A low infant exposure, approximately 3.34% of the daily maternal dose and approximately 2.26% of the recommended daily infant dose, was estimated.

Breast milk exposure has widely been reported for several first‐line antiretroviral drugs.^17^ Plasma‐to‐breast milk transfer of lamivudine has been described in several studies.^6^, ^7^, ^8^, ^9^, ^10^, ^11^ Most studies describe the breast milk transfer based on paired plasma–breast milk samples obtained at single timepoints^6^, ^7^, ^8^, ^9^ in contrast to longitudinal concentration‐time profiles.^10^, ^11^ Most studies have reported breast milk accumulation of lamivudine with a milk‐to‐plasma ratio >1. The variability in estimated milk‐to‐plasma ratio is largely driven by the timepoints of sampling and caution should be taken during the interpretation: the milk‐to‐plasma ratio of 1.77 (IQR: 1.64, 1.87) in this study was based on plasma and breast milk concentrations across a 24‐h dosing interval, compared to 1.21 (IQR: 1.06, 1.57) based on exposure between 0 and 6 h,^10^ 0.95 (0.82–1.15) based on exposure between 0 and 12 h,^11^ and 3.04 (2.87–4.16) based on exposure between 12 and 20 h.^11^ Generally, the steady‐state AUC across the entire dosing interval provides the best comparative measure of plasma and breast milk exposure accounting for all pharmacokinetic processes. A lag in time of transfer of lamivudine from maternal plasma‐to‐breast milk observed in this study was also previously reported.^7^, ^10^

This work provides the first lamivudine lactation population pharmacokinetic model. In a preliminary analysis of the same data, the breast milk transfer was described as a fraction of the total plasma clearance with the breast milk volume fixed to a physiological value.^28^ In the current analysis, the effect compartment concept^18^ was used allowing characterization of the delayed plasma‐to‐breast milk distribution of lamivudine and the milk‐to‐plasma ratio. Interindividual variability was implementable on the volume of distribution, plasma clearance, and milk‐to‐plasma ratio but not the absorption rate constant. Twenty‐one of the 35 women lacked plasma concentration data in the absorption phase leading to poor precision in parameter estimate and high shrinkage.^29^ We could not estimate precisely the covariate effects of creatinine clearance on plasma clearance contrary to earlier publications that showed a statistically significant association between creatinine clearance and lamivudine clearance.^30^, ^31^, ^32^ This observation is possibly attributed to the significantly low number of patients. Population pharmacokinetic analysis simultaneously characterizes the longitudinal changes in drug concentrations across time, the within‐ and between‐patient variability, and further facilitates estimation of individual plasma and breast milk exposure over 24 h for computation of the milk‐to‐plasma ratio.

The clinical implications of infant exposure to antiretroviral drugs through breast milk are not fully understood. Metrics of relative exposure to daily maternal‐ or infant doses are often reported as references for drug safety.^33^, ^34^ The estimated RID of 3.34% from this study is lower than the 10% threshold^33^, ^34^ commonly referenced for drug safety in breastfeeding mothers and the estimated exposure of 2.26% relative to the daily infant dose is also lower than the 6% reported for lamivudine in breastfeeding infants.^35^ However, high rates of resistance to lamivudine have been reported in infants who acquire HIV through breast milk while their mothers are receiving lamivudine, even at these low exposure levels,^13^, ^15^ which likely relates to infant concentrations below the recommended therapeutic concentration, thereby allowing selection of resistant mutants.

The dynamic time‐course of infant exposure could not be evaluated from the current dataset. Infant exposure is dependent on several factors including the volume of breast milk ingested, breastfeeding frequency, time of breastfeeding relative to maternal dosing, infant age, drug bioavailability, and disposition in the infant,^33^ most of which were not explicitly documented in this study. However, the estimated infant steady‐state concentrations approximate the observed infant concentration ranges and were similar to previously reported lamivudine infant concentrations (Table 3).^7^, ^8^, ^10^, ^11^, ^16^

**TABLE 3:** Pharmacokinetic studies measuring infant plasma concentrations of lamivudine from exposure through breast milk in mothers receiving lamivudine‐based antiretroviral therapy.

Study	Number of infants	Lamivudine dose	Infant feeding	Infant plasma sampling time	Infant plasma concentrations
Mirochnick L et al., 2009^7^	67	150 mg bd	–	24 h, 2, 6, 14, and 24 weeks after delivery	Median (IQR) 24 h postpartum: 67 ng/mL (48–168) Week 2: 32 ng/mL (23–44) Week 6: 24 ng/mL (16–40) Week 14: 20 ng/mL (15–29)
Palombi L et al., 2012^8^	66	150 mg bd	–	At 1, 3 and 6 months postpartum: Specific times of the day not reported	Median (IQR) Month 1: 27 ng/mL (14–57) Month 3: 12.5 ng/mL (6.8–30.4) Month 6: 2.5 ng/mL (2.5–18.9)
Corbett AH et al., 2014^10^	30	150 mg bd	Mothers freely breastfed during the study period	Either week 6, 12, 24 post‐partum. Infant plasma was sampled prior to maternal dose; at 2, 4, and 6 h post maternal dose	Average concentration: 0.018 μg/mL (0.010–0.0280)
Waitt C et al., 2018^11^	30	150 mg bd and 300 mg od	Mothers freely breastfed during the study and the time of breastfeeding was recorded	All infants were <6 months of age. Infant plasma sampling was performed at 2, 4, 14, 18 h postdose	Lamivudine detectable in the plasma of 36% of the infants. Median (range): 17.7 μg/mL (16.3–22.7)
Shapiro RL et al., 2005^16^	20	150 mg bd	0–3 h	2 months postpartum (for 13 infants); 5 months post‐partum (for 7 infants)	Median: 28 ng/mL

As limitations, our data did not support the evaluation of change in lamivudine plasma‐to‐breast milk transfer across time. The times of the first study (pharmacokinetic sampling) visit widely varied across patients and overlapped with that of the second study visit which limits the independent evaluation of pharmacokinetics within a visit. However, as a strength, this study describes the lactation pharmacokinetics of lamivudine across a long post‐partum time frame (1–12 weeks) accounting for several time‐varying maternal and infant factors. Secondly, many women were dosed at night, meaning that we relied on self‐reported dosing times and could not obtain pharmacokinetic samples at early timepoints. This challenge was attributed to: (i) the lack of facilities to do pharmacokinetic sampling at night, (ii) our intent not to disrupt the normal dosing times which could potentially disrupt adherence at what is known to already be a time of risk for suboptimal adherence.^36^ Third, infant feeding patterns (time schedule and quantity of breast milk consumed) during PK sampling was not explicitly documented or standardized across study participants, potentially introducing variability in measured breast milk and infant drug concentrations. This also limited the possibility to explicitly model the infant data. We have previously found that collecting this data with accuracy was very difficult due to erratic infant feeding patterns that make it impossible to ascertain the beginning or end of the feed. Lastly, steady‐state pharmacokinetics was assumed but not explicitly evaluated. Mothers were recruited antenatally and the earliest pharmacokinetic sample taken on day 7 post‐partum, a sufficient time period to have attained steady‐state concentrations. This assumption may however not account for the impact of missed doses.

In conclusion, a population lactation pharmacokinetic model of lamivudine was developed describing the time lag in plasma‐to‐breast milk drug transfer and breast milk drug accumulation. The model predicted a low daily infant lamivudine breast milk exposure which approximated the observed infant steady‐state concentration in a freely breastfed infant. The established framework can be extended to characterize the plasma‐to‐breast milk transfer across different therapeutic areas.

## AUTHOR CONTRIBUTIONS

F.W.O., A.N.K., R.N., and C.W. wrote the manuscript; C.W. designed the research; S.N., I.K., M.L., S.K., H.P., and C.W. performed the research; F.W.O. analyzed the data. All authors read and approved the submission of this manuscript to CPT: Pharmacometrics and Systems Pharmacology.

## FUNDING INFORMATION

F.W.O, A.N.K, and R.N were supported by a Wellcome Trust Clinical Research Career Development Fellowship awarded to Catriona Waitt 222075_Z_20_Z. S.N and IK were supported by Wellcome Postdoctoral Training Fellowship for Clinicians 104422/Z/14/Z awarded to CW.

## CONFLICT OF INTEREST STATEMENT

The authors declared no competing interests for this work.

## Supporting information


Data S1.

Figure S1.

Table S1.
