# Supplementary material for: Population pharmacokinetic modeling of paired plasma–breast milk lamivudine data for estimation of infant exposure in breastfeeding mother–infant pairs
Source: CPT Pharmacometrics Syst Pharmacol. 2024 Nov 7;13(11):1978–89. doi: 10.1002/psp4.13274 (PMC11578128; doi:10.1002/psp4.13274)
Supplement: Supplementary file 1 — Data S1. [file PSP4-13-1978-s001.docx]

**Model code characterizing the lactation pharmacokinetics of lamivudine**

$INPUT ID TIME TAD OCC AMT ADDL II EVID MDV CMT DV REG WT HT AGE BWT CREAT CRCL

$DATA data_3TC_NONMEM_BMPLASMA4_rate.csv IGNORE=#

$SUBROUTINES ADVAN13 TOL=6

$MODEL

NCOMP=3

COMP=DEPOT

COMP=CENTRAL

COMP=EFFECT

$PK

CL= THETA(1)*EXP(ETA(1)) ; Plasma clearance of the central compartment

VC= THETA(2)*EXP(ETA(2)) ; Volume of distribution of the central compartment

KA= THETA(3)*EXP(ETA(3)) ; First-order absorption rate constant

KCB= THETA(4)*EXP(ETA(4)) ; Plasma and breast milk equilibration rate constant

RCB= THETA(5)*EXP(ETA(5)) ; Lamivudine breast milk accumulation rate constant

S2=V2

$DES

DADT(1) = -KA*A(1)

DADT(2) = KA*A(1)-(CL/VC)*A(2)

DADT(3) = KCB*(RCB*A(2)/VC-A(3))

$ERROR

IF(CMT.EQ.2) Y=F*(1+EPS(1))

IF(CMT.EQ.3) Y=F*(1+EPS(2))

$THETA

19.4 ; THETA(1)

184 ; THETA(2)

1.87 ; THETA(3)

0.245 ; THETA(4)

1.77 ; THETA(5)

$OMEGA BLOCK (2) 0.0435 0.0671 0.584

$OMEGA (0 FIX) ; IIV KA

$OMEGA (0 FIX) ; IIV KCB

$OMEGA (0.0252) ; IIV RCB

$SIGMA

(0.147) ; PROP RUV PLASMA

(0.093) ; PROP RUV BM
