# Supplementary material for: Population pharmacokinetic modeling of paired plasma–breast milk lamivudine data for estimation of infant exposure in breastfeeding mother–infant pairs
Source: CPT Pharmacometrics Syst Pharmacol. 2024 Nov 7;13(11):1978–89. doi: 10.1002/psp4.13274 (PMC11578128; doi:10.1002/psp4.13274)
Supplement: Supplementary file 2 — Figure S1. [file PSP4-13-1978-s003.docx]

**Title: Population pharmacokinetic modelling of paired plasma-breast milk lamivudine data for estimation of infant exposure in breastfeeding mother-infant pairs**

**Authors:** Francis Williams Ojara^1,2^, Aida N Kawuma^1^, Shadia Nakalema^1^, Isabella Kyohairwe^1^, Ritah Nakijoba^1^, Mohammed Lamorde^1^, Henry Pertinez^3^, Saye Khoo^3^, *Catriona Waitt^1,3^

**Affiliation:**

1Infectious Diseases Institute, Makerere University College of Health Sciences, Uganda

2Department of Pharmacology and Therapeutics, Gulu University, Uganda

3Department of Pharmacology and Therapeutics, University of Liverpool, UK

***Corresponding author**

Dr. Francis Williams Ojara

Infectious Diseases Institute, Makerere University College of Health Sciences, Uganda / Department of Pharmacology and Therapeutics, Gulu University, Uganda

Email: francisojarawilliams@yahoo.com


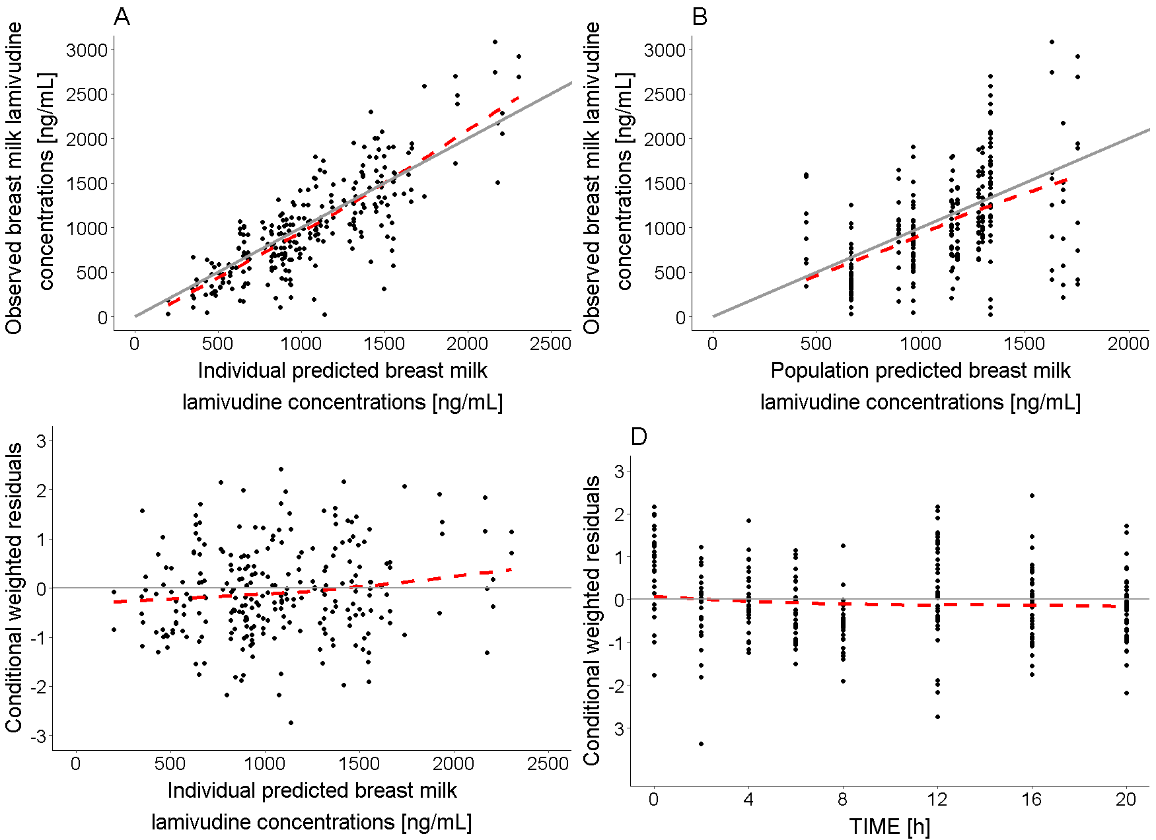


**Figure S1.** Goodness-of-fit for lamivudine breast milk data from the population pharmacokinetic model characterizing the maternal plasma-to-breast milk transfer of lamivudine. Solid circles: data points; solid grey line: Line of identity; dashed red line: Trend line for the observed data; Plots, A: observed versus individual predicted lamivudine concentrations; B: observed versus population predicted lamivudine concentrations; C: conditional weighted residuals versus individual predicted lamivudine concentrations; D: conditional weighted residuals versus time.
