# Supplementary material for: Population pharmacokinetic modeling of paired plasma–breast milk lamivudine data for estimation of infant exposure in breastfeeding mother–infant pairs
Source: CPT Pharmacometrics Syst Pharmacol. 2024 Nov 7;13(11):1978–89. doi: 10.1002/psp4.13274 (PMC11578128; doi:10.1002/psp4.13274)
Supplement: Supplementary file 3 — Table S1. [file PSP4-13-1978-s002.docx]

**Table S1.** Parameter estimates of the population pharmacokinetic model characterizing the plasma disposition of lamivudine

| **Parameter [Units]** | **Estimates (%RSE)** | |
| --- | --- | --- |
|  | **Base model**  **(No covariates)** | **Covariate model** |
| CL [L•h^-1^] | 20.6 (4.2) | 20.0 (10.3) |
| VC [L] | 193 (11.9) | 173 (31.5) |
| Ka [h^-1^] | 2.27 (20.8) | 2.03 (27.8) |
| CLCR on CL | -- | 0.0998 (31.5) |
| WT on VC | -- | 5.64 (48.2) |
| IIV CL, % CV | 17.9 (15.3) | 12.4 (34.2) |
| IIV VC, % CV | 52.0 (36.7) | 49.1 (35.1) |
| IIV Ka, % CV | 0 FIX | 0 FIX |
| RUV, PROP, PLASMA, % CV | 42.4 (6.1) | 41.2 (8.9) |

CL: Plasma clearance; VC: Volume of distribution of the central compartment; Ka: 1^st^ – order absorption rate constant; CLCR: Creatinine clearance; IIV: Interindividual variability; CV: coefficient of variation; RUV: Random unexplained variability; RSE: Relative standard error.
